# Preterm infant with diprosopus and holoprosencephaly

**DOI:** 10.1002/ccr3.5163

**Published:** 2021-12-22

**Authors:** Nitya M. Nair, Daniel T. Swarr, Maria E. Barnes‐Davis

**Affiliations:** ^1^ Division of Neonatology Department of Pediatrics Emory University School of Medicine and Children’s Healthcare of Atlanta Atlanta Georgia USA; ^2^ Perinatal Institute Section of Neonatology Cincinnati Children’s Hospital Medical Center Cincinnati Ohio USA; ^3^ Department of Pediatrics University of Cincinnati College of Medicine Cincinnati Ohio USA

**Keywords:** conjoined twinning, craniofacial duplication, diprosopus, holoprosencephaly

## Abstract

Diprosopus is an extremely rare congenital anomaly involving craniofacial duplication. The etiology and pathophysiology remain unknown, and no genetic mutations have been definitively associated with the condition. This case describes an infant born at 27‐weeks completed gestation with multiple congenital anomalies including diprosopus and discusses the implications of prenatal diagnosis.

## 1 INTRODUCTION

Diprosopus is an extremely rare congenital anomaly involving partial or complete craniofacial duplication in the context of a single trunk and phenotypically normal limbs.^1^ It is considered a subtype of symmetric monocephalic conjoined twinning, and less than 40 cases have been reported in the literature to date.^2^, ^3^ Although diprosopus is seen more commonly in animals such as cattle, pigs, and cats, its occurrence is much less frequent in humans.^4^ Conjoined twinning is estimated to occur with a prevalence of 1.47 (95% CI: 1.32–16.2) per 100,000 births.^5^ Diprosopus, considered by some to be the rarest form of conjoined twinning, has a prevalence of 1 in 15 million births. Stillbirths occur in 50% of all conjoined twins, and the majority of infants born alive do not survive long‐term.^4^, ^6^ Diagnosis can be made on prenatal ultrasound imaging, and prognosis is variable based on the presence or absence of associated clinical features. Complete facial duplication, which involves the duplication of at least two full facial organs or two structures from two different organs in an individual with one head and one trunk, is commonly associated with other congenital anomalies including the central nervous system, the cardiovascular system, the gastrointestinal system, and the respiratory system.^2^, ^7^ Prognosis for this rare condition is typically poor. However, partial facial duplication, defined as duplication of only a single organ or part of an organ from the face of an individual with one head and one trunk, is associated with fewer co‐existing anomalies, and, therefore, has a more favorable overall prognosis with surgical amelioration possible in some cases.^2^, ^8^

In this short report, we present the case of an infant girl born at 27‐weeks of completed gestation with a postnatal diagnosis of diprosopus and multiple associated congenital anomalies. We will discuss possible etiologies and pathophysiological mechanisms with the ultimate aim of educating providers who might encounter these infants. Informed consent for this case report was obtained from the infant's mother.

## 2 CASE PRESENTATION

A 29‐year‐old gravida 2 para 1 Black woman presented for prenatal care at 16‐week gestation. Her first pregnancy was reportedly uncomplicated, and she delivered a healthy term infant. Prenatal ultrasound during this pregnancy was concerning for abnormal midline brain and facial structures including difficult‐to‐visualize cisterna magna, cavum septa pellucidi, facial profile, nose, and lips. A detailed anatomy scan was recommended at that time but was not obtained. Notably, there was no history of consanguineous marriage or family history of congenital anomalies such as holoprosencephaly.

## 3 CLINICAL FINDINGS AND TIMELINE

A 1090‐g infant girl was delivered via emergent cesarean section at 27‐week and 3‐day gestation in the context of preterm labor and breech presentation. The mother had significant polyhydramnios, and the infant was immediately noted to have significant craniofacial malformations that were not anticipated by the parents or the care team. On examination, the infant was phenotypically female with grossly normal anatomy of the trunk and extremities. She had no spontaneous movements. Facial structures were difficult to define at the time of birth. She had two well‐formed but very small mouths with corresponding mandibular structures. However, these structures were too small to allow passage of endotracheal tubes. Two protuberant tubular structures were in the expected locations of the eyes, and a hairy midline mass had no definable airway structures. Her initial heart rate was low. Positive pressure ventilation was applied over the two mouth‐like structures. However, aeration on auscultation was poor, and there was no improvement in heart rate. During the resuscitation, air was noted to exit through the tubular structures in the expected location of the eyes. The care team discussed the differential diagnosis and suspected the infant had diprosopus. After sharing this with the parents and inquiring regarding goals of care, parents reiterated they had not anticipated this degree of congenital anomaly and that the infant was a full code. Chest compressions were initiated, and umbilical line placement was prepared for fluid resuscitation and epinephrine administration. At nine minutes of life, parents asked for resuscitative efforts to stop, and care was redirected to comfort measures. The care team was supportive of this decision. Cord arterial gas showed a pH of 6.9 and a base deficit of 16. Apgar scores were 2 and 2 at one and five minutes of life, respectively. The infant died shortly after birth.

## 4 DIAGNOSTIC INVESTIGATION AND OUTCOME

**FIGURE 1:**
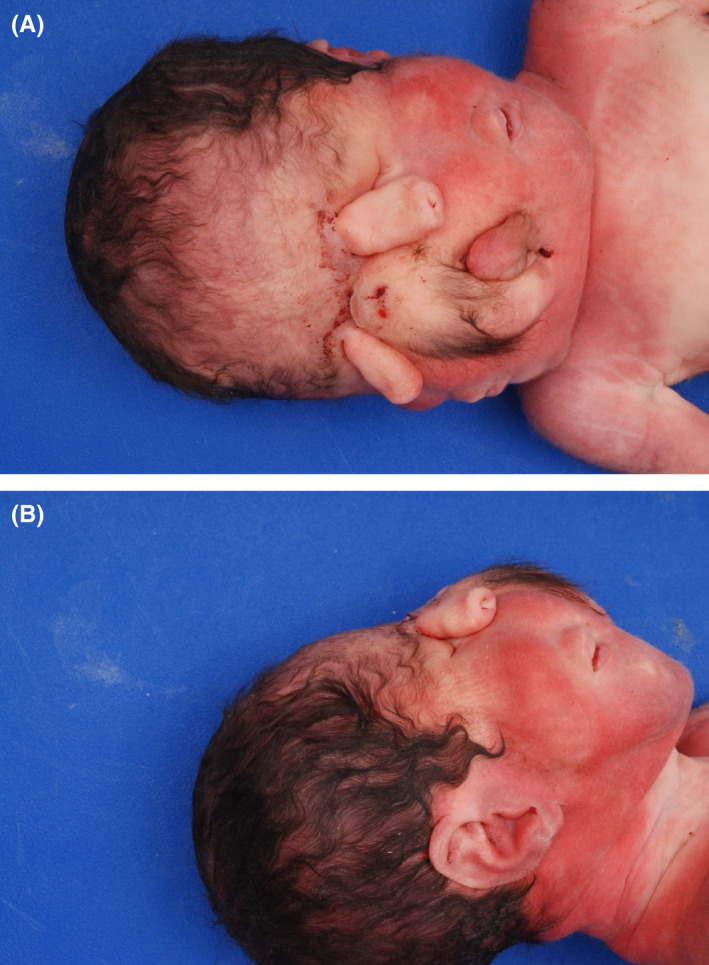
External facial anomalies. Panel A. Anterior view of face with two proboscides above rudimentary eye structures, two mouths, and a midline hairy mass consistent with ectopic scalp tissue. Panel B. Facial profile view with one of three ears, right mouth, and right proboscis

**FIGURE 2:**
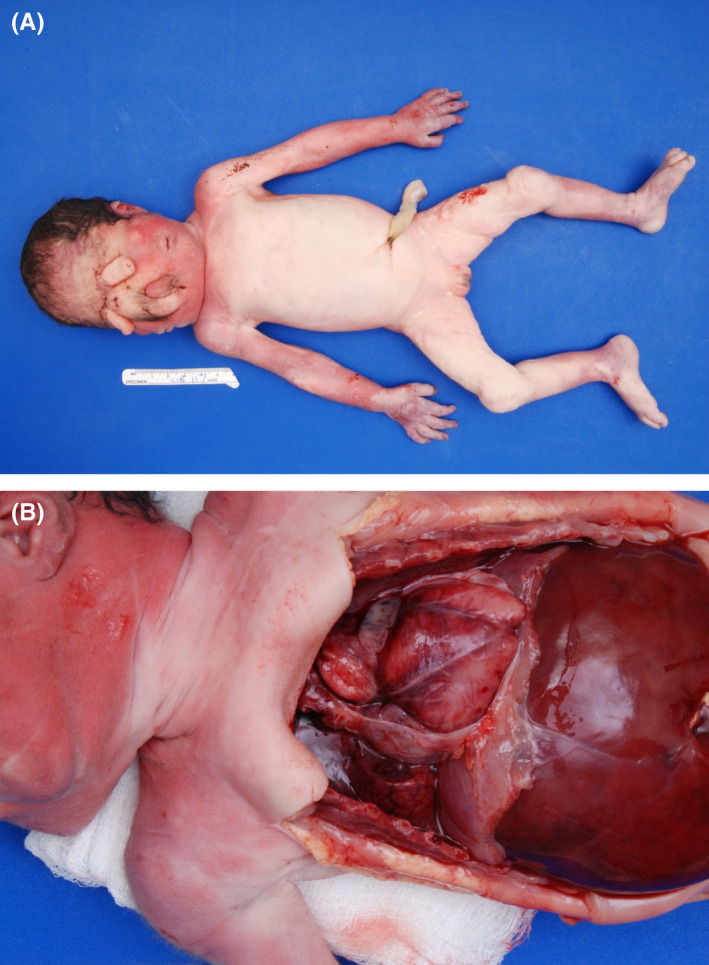
Full body and internal views. Panel A. Full body view of infant with external female genitalia, single trunk, and duplicated facial structures. Panel B. Internal view of chest and abdomen including heart with bifid apex and hepatomegaly

Parents consented to autopsy and expressed a desire for genetic testing as a way to obtain closure following the death of their daughter and to guide further family planning. Autopsy was notable for incomplete facial duplication with three ears; two tubular proboscides above rudimentary eye structures (left microphthalmia and right anophthalmia); two mouths which did communicate with an oropharyngeal cavity and a single midline trachea; and a midline hairy mass consistent with invagination of the scalp (Figure 1). Nervous system findings were notable for microcephaly with absent fontanelles, the aforementioned primitive eye structures, and holoprosencephaly. While the trunk of the infant appeared phenotypically typical on external examination, several congenital anomalies were noted upon dissection. These included a heart with a bifid apex and two anterior descending coronary arteries, two splenules, hepatomegaly, pulmonary hypoplasia, and a proximal umbilical cord stricture (Figure 2). Karyotype showed 46, XX, and no pathogenetic deletions or duplications were identified on microarray. As part of research testing for the infant and both biological parents, a holoprosencephaly panel was performed by the NIH Medical Genetics Diagnostic Laboratory, which included the genes SHH, ZIC2, SIX3, and TGIF1. However, no pathogenic variants were identified. The case was discussed with an expert pediatric otolaryngologist postnatally in response to questions from the family, and they confirmed that the severity of this infant's abnormalities would not have been amenable to surgical intervention or airway reconstruction. The care team followed up with the parents after the autopsy results were known and before the writing of this report to answer questions and offer support to the family.

## 5 DISCUSSION

Diprosopus is a rare congenital anomaly involving craniofacial duplication that has been associated with conditions affecting the central nervous system (brain duplication, anencephaly, holoprosencephaly, Chiari II malformation, and neural tube defects); the cardiovascular system (Tetralogy of Fallot, laterality disorders); the pulmonary system (congenital diaphragmatic hernia); the gastrointestinal system (laterality disorders, malrotation); the genitourinary system (multicystic dysplastic kidney disease, horseshoe kidney); and the musculoskeletal system (spinal anomalies).^2^, ^9^ Our case involved many of these systems with holoprosencephaly, pulmonary hypoplasia, cardiac defects, and gastrointestinal anomalies including hepatomegaly and two splenules. The finding of possible ectopic scalp tissue seen on the midline of the face is unique and has not been reported in the literature to date.

In a systematic review of diprosopus case reports, Bidondo et al. identified 31 patients reported in the literature, 15 born alive, 5 stillbirths, 9 electively terminated, and 2 without outcomes available. They found a female predominance in affected infants with a mean gestational age of 34.5 weeks and cited the most frequently duplicated structures as the nose and eyes. The most commonly associated anomalies were those involving the central nervous system (up to 45%), the cardiovascular system (up to 53%), and laterality defects (up to 37%).^2^ It is rare for children with complete diprosopus to survive more than a few hours, but for those with incomplete diprosopus who are able to survive, surgical intervention is often needed for functional and esthetic purposes.^6^ While it is difficult to place this reported case in the context of other cases of diprosopus due to the rarity and heterogeneity of cases, our case is congruent with the literature in that the affected infant was female, she had duplication and malformation of upper airway features, and she had associated systemic congenital anomalies. The tubular upper airway structures, ectopic scalp tissue, and live birth despite extent and severity of anomalies are unique.

The exact etiology and pathophysiology of diprosopus remain unknown, although it is likely that they are multifactorial.^6^ The traditional model of monozygotic twinning involves the product of a single ovum and sperm that divides to form two embryos, while alternative models suggest that embryonic fusion events underlie this process.^10^ Several proposed pathogenic mechanisms for diprosopus exist, including 1) early ventrolateral fusion of two monozygotic embryonic disks with reorganization of merged tissues leading to secondary aplasia and divergence of tissues from the midline; 2) neurocristopathy with fission in a monozygotic gestation that produces two notochords that then generate a duplicated cephalic neural plate, an extra medial cranial neural crest, and alterations in the paraxial mesoderm; and 3) two early primitive nodes in a single embryo prior to the formation of the notochord.^2^

In contrast to the theories regarding atypical twinning, Hu et al. investigated the role of Sonic hedgehog (SHH), an important protein in craniofacial patterning during development that defines the mediolateral axis of the embryo.^4^ In avian models, it was found that transient loss of SHH signaling inhibits growth of the primordia and results in defects such as hypotelorism and cleft lip or palate while excess SHH leads to mediolateral widening of the frontonasal process and hypertelorism, which when severe, can lead to facial duplications.^11^ Interestingly, SHH has been implicated in the pathogenesis of some types of holoprosencephaly.^12^, ^13^ Given the published association between diprosopus and laterality defects, one could also speculate a ciliopathy and disruption of other genes associated with left‐right patterning defects may contribute to the pathogenesis of this disorder. Interestingly, the mother of this patient had a subsequent twin pregnancy in which one of the fetuses had a meningomyelocele. Although it is unclear if there is a genetic link between spina bifida and diprosopus, it could also be speculated that aberrant SHH signaling as theorized in the pathogenesis of diprosopus could be related to that which is implicated in the pathogenesis of spina bifida.^14^

To date, no genetic mutations have been definitively associated with diprosopus, which continues to support an embryologic theory of abnormal twinning.^9^ Some case reports identify mutations of unknown significance, but none have been robustly linked to this disorder. Our patient had a karyotype and microarray, and no pathogenetic deletions or duplications were identified on either study. Further genetic testing of the infant and parents included a holoprosencephaly panel, which did not identify any genetic mutations in the SHH, ZIC2, SIX3, or TGIF1 genes.

## 6 CONCLUSION

Diprosopus is an extremely rare condition of unknown etiology, which is usually fatal. Further research will be paramount to elucidating the pathophysiology of this unique diagnosis and could contribute to our understanding of the mechanisms involved in typical and atypical twinning. We also anticipate that high‐throughput sequencing studies of affected infants may provide future insights into whether disruption of the SHH signaling pathway, cilia function, or other genes involved in left‐right patterning may contribute to the pathogenesis of diprosopus. A greater awareness of this condition and its underlying etiology would allow families and care teams the opportunity to develop a comprehensive care plan prior to delivery. Moreover, the fact that the family reported here subsequently had a pregnancy impacted by a fetus with a neural tube defect highlights the importance of comprehensive prenatal counseling and genetic testing, as indicated, for any pregnancy impacted by a major congenital malformation such as diprosopus. Increased awareness and antenatal counseling for patients impacted by diprosopus can improve care provided to the affected infant and aid in the support and counseling offered to the family. Comfort care should be offered to affected families as an option with palliative care available at the time of birth, and full neonatal resuscitation teams and advanced life support capabilities should be available at delivery for infants of parents who desire resuscitation. Improving our management and counseling is vital to improving the care and quality of life experienced by these infants and their families.

## CONFLICTS OF INTEREST

The authors have no conflicts of interest to report.

## AUTHOR CONTRIBUTIONS

NN contributed to the collection of data, analysis of results, and writing of the manuscript. DS contributed to the writing and critical revision of the manuscript. MBD contributed to the care of this family, collection of data, analysis of results, and the writing and critical revision of the manuscript.

## ETHICAL APPROVAL

As this is a case report, it does not meet criteria for human subject research, and thus, approval by the Institutional Review Board and written informed consent are not required. The local practice, though, is that when a rare diagnosis is involved or there is a reasonable likelihood that patients could be identified, that family is contacted regarding intent to report the scientific details of the case. In this instance, informed consent was obtained from the mother, and she approved of the manuscript in its written form.

## CONSENT

Patient perspective was unable to be obtained for this infant. However, the patient's family reviewed the case report, and written consent was obtained from the patient's family to report this case.
